# SENIOR HOUSINGS AND COMMUNITY FOR SOCIAL CONNECTION AND HEALTH AMONG LOW-INCOME OLDER ADULTS

**DOI:** 10.1093/geroni/igae098.1959

**Published:** 2024-12-31

**Authors:** Sojung Park, Robyn Stone

**Affiliations:** Chair; Discussant

## Abstract

The symposium aims to enhance the quality of life for low-income seniors in community-based housing in the US. The focus is on social connection, neighborhood resources, and community partnerships. Analyzing the 12-year-long national data, the first study used the synthetic difference in difference regression to compare social isolation and cognitive function in two groups of older individuals aged 70 and over. Residents in traditional housing were more likely to experience increased social isolation and decreased cognitive function than senior housing residents. Senior housing was protective, particularly for low-income older adults. A qualitative study on subsidized senior housing (SSH) in Baltimore City found some residents had a small network of connections; most were cordial but disconnected. Challenges related to aging, undesirable behaviors of others, and a desire for privacy limited social connections led to social isolation within the housing communities. Using A Geographic Information System approach, another study looked at SSH availability in New Jersey and its link to neighborhood disadvantage. SSH tends to be located in areas experiencing more significant economic deprivation, highlighting the need for government intervention to mitigate geographic disparities in SSH provision. The last study presented a case of Senior Housing Preservation-Detroit to address the displacement of older adults and improve the quality of life in senior housing. The presentation will cover COVID-19 outreach, lessons learned, milestones, and an emergency plan for Detroit’s older population. Our studies provides a rigorous basis for future research to identify factors and mechanisms for healthy aging among low-income senior housing residents.

